# Supplementary material for: WordCommentsAnalyzer: A windows software tool for qualitative research
Source: F1000Res. 2018 Sep 4;7:536. Originally published 2018 May 3. [Version 2] doi: 10.12688/f1000research.14819.2 (PMC6137411; doi:10.12688/f1000research.14819.2)
Supplement: Supplementary file 3 [file f1000research-7-17614-s0002.tgz › b2a79496-4ada-47c4-9682-a99ce867836d.zip!/Eight Iranian Health Journals/Salmand_Iranian Journal of Ageing _11.docx]

© Salmand_Iranian Journal of Ageing . This work is licensed under CC BY-NC 4.0 (https://creativecommons.org/licenses/by-nc/4.0/).

Volume & Issue: دوره 10 - شماره 3

Title: بررسی نیازهای فرهنگی، اجتماعی، اقتصادی، رفاهی و ساختارِ شهری اعضای کانون سالمندان شهر تهران

http://salmandj.uswr.ac.ir/article-1-686-fa.html

اهداف: هدف از این مطالعه شناسایی نیازهای فرهنگی، اجتماعی، اقتصادی، رفاهی و ساختار ِشهری اعضای کانون سالمندان شهر تهران و شناخت وضعیت فعلی آن‌ها در 8 حیطه با رویکرد به پروژه سازمان جهانی بهداشت در مورد شاخص‌های شهر دوستدار سالمند است. مواد و روش‌ها: در پژوهش توصیفی-استنباطی، 400 سالمند ساکن شهر تهران و عضو کانون سالمندان (276 نفر زن و 124 نفر مرد با میانگین سنی 63 سال) با روش نمونه‌گیری تصادفی در پژوهش شرکت کردند. داده‌ها با پرسش‌نامه مشتمل بر دو بخش توصیفی و استنباطی جمع‌آوری شد. بخش توصیفی خود به دو بخش ویژگی‌های دموگرافیک (شامل 12 سؤال) و نیازهای سالمندان در 8 حیطه و 75 شاخص تقسیم شد. شاخص‌ها در بازه نمره 5-1 مورد ارزیابی قرار گرفتند و تجزیه و تحلیل داده‌ها با نرم‌افزار SPSS انجام شد. یافته‌ها: در حیطه بناها و فضاهای باز بیشترین نمره به «تمیزی محوطه‌های عمومی» و کمترین نمره به «ارائه خدمات ویژه در فروشگاه‌ها و بانک‌ها» (با نمره میانگین 2/50 و 1/65)، در حیطه حمل‌و‌نقل بیشترین نمره به «مناسب‌بودن محل نصب چراغ‌های راهنمایی در تقاطع‌ها» و کمترین نمره به «حمل‌ونقل ویژه سالمندان» (با نمره میانگین 3/03 و 1/58)، در حیطه مسکن بیشترین نمره به «مناسب‌بودن طراحی داخلی خانه‌ها» و کمترین نمره به «مسکن ارزان‌قیمت و به تعداد کافی» (با نمره میانگین 1/93 و 1/51)، در حیطه مشارکت اجتماعی بیشترین نمره به «زمان مناسب برگزاری مناسبت‌های ویژه سالمندان» و کمترین نمره به «امداد به سالمندانِ در معرض انزوای اجتماعی» (با نمره میانگین 2/88 و 2/07)، در حیطه تکریم سالمندان و اجتماع‌پذیری اجتماعی بیشترین نمره به «نگاه مثبت به سالمندان در رسانه‌های جمعی» و کمترین نمره به «مشورت با سالمندان درمراکز تجاری برای ارائه خدمات بهتر» (با نمره میانگین 2/84 و 1/74)، در حیطه مشارکت شهروندی و اشتغال بیشترین نمره به «آموزش کارکنان سالمند برای مشاغل بعد از بازنشستگی» و کمترین نمره به «حمایت از فرصت‌های خوداشتغالی برای سالمندان» (با نمره میانگین 2/87 و 1/75)، در حیطه اطلاعات و ارتباطات بیشترین نمره به «امکان برقراری ارتباط کلامی» و کمترین نمره به «دسترسی عمومی و رایگان سالمندان به رایانه و اینترنت» (با نمره میانگین 2/33 و 1/77) و در حیطه حمایت اجتماعی و خدمات سلامت بیشترین نمره به «حمایت و ترغیب خدمات داوطلبانه» و کمترین به «شامل‌بودن هر دو نوع خدمات خانه‌داری و مراقبت‌های بهداشتی در خدمات مراقبتی خانگی» (با نمره میانگین 2/23 و 1/78) اختصاص یافت. نتیجه‌گیری: در حیطه‌های هشت‌گانه، میانگین ارزیابی سالمندان از میزان برخورداری از نیازها کمتر از حد میانگین (2/11) بوده است. با کمی چشم‌پوشی تنها حیطه «مشارکت اجتماعی» به ‌مرز متوسط نزدیک شده و حیطه «مسکن» کمترین نمره را به‌دست آورده است. به‌عبارت دیگر، برخورداری سالمندان از مسکن بیشترین نیاز در بین دیگر نیازها بوده است.

Title: بررسی اثر تمرینات یوگا بر آمادگی جسمانی و تعادل زنان سالمند

http://salmandj.uswr.ac.ir/article-1-799-fa.html

اهداف: شروع سن سالمندی و کاهش فعالیت بدنی، باعث ایجاد اثرات نامطلوب فیزیکی و روانی در افراد سالمند می‌شود. ازآنجایی‌که فعالیت بدنی می‌تواند باعث بهبود وضعیت جسمانی و حتی بهبود وضعیت روانی شود و به‌دنبال آن استقلال بیشتر در افراد سالمند به‌وجود آورد، هدف از این مطالعه تعیین اثر برنامه هاتا یوگا بر برخی عوامل آمادگی جسمانی سالمندان بود. مواد و روش‌ها: 45 زن سالمند با انحراف استاندارد 6/12±64 سال، 10/21±162/5 سانتی‌متر و 9/71±63/31 کیلوگرم که از سلامت عمومی برخوردار بودند، به‌صورت نمونه‌گیری دردسترس برای شرکت در این مطالعه انتخاب شدند. در این تحقیق برای اندازه‌گیری تعادل ایستا از آزمون شارپندرومبرگ (با چشمان باز و بسته)، تعادل پویا از آزمون عملکردی دستیابی، چابکی از آزمون TUG، قدرت اندام فوقانی تعداد خمیدگی بازو در زمان 30 ثانیه و اندام تحتانی تعداد نشستن و برخاستن از صندلی در زمان 30 ثانیه، انعطاف‌پذیری اندام فوقانی از آزمون رساندن دست‌ها از پشت به‌هم و برای انعطاف‌پذیری اندام تحتانی از آزمون خمش و رسش روی صندلی استفاده شده است. آزمون‌ها قبل و بعد از برنامه تمرینی اجرا شد. تحلیل آماری داده‌ها به‌وسیله آزمون تحلیل واریانس و تی همبسته (در سطح معناداری 0/05>P) صورت گرفت.  یافته‌ها: یافته‌های پژوهش حاضر نشان داد که 6 هفته تمرینات یوگا بر تعادل ایستا با چشمان باز و بسته، تعادل پویا، چابکی، قدرت عضلانی اندام فوقانی و تحتانی، انعطاف‌پذیری اندام فوقانی و تحتانی تأثیر معنی‌داری دارد. نتیجه‌گیری: به‌نظر می‌رسد تمرینات یوگا (هاتایوگا) بر آمادگی جسمانی سالمندان تأثیرات مثبت می‌گذارد. این تأثیرات شامل بهبود تعادل ایستا و پویا، چابکی، قدرت در اندام فوقانی و تحتانی و انعطاف‌پذیری اندام فوقانی و تحتانی است.

Title: آیا تعادل ایستا و پویای سالمندان مرد سالم در اوقات مختلف روز متفاوت است؟

http://salmandj.uswr.ac.ir/article-1-654-fa.html

اهداف: عملکردهای تعادلی در زندگی روزمره سالمندان به‌­ویژه در کاهش خطر افتادن آنها نقشی مهم ایفا می‌­کند. هدف پژوهش حاضر، بررسی تأثیر اوقات روز بر تعادل ایستا و پویای سالمندان مرد سالم بود. مواد و روش‌ها: در این مطالعه، ده مرد با میانگین سنی 3/23±69/45 سال به‌طور داوطلب در مطالعات شرکت کردند. در ساعات 8، 12، 16 و 20، عملکردهای تعادلی ایستا و پویای آزمودنی‌ها با استفاده از آزمون لک‌لک و آزمون گردش ستاره‌ای اندازه‌گیری شدند. یافته‌ها: نتایج آزمون تحلیل واریانس بر عدم معناداری تأثیرات اوقات روز بر تعادل ایستا با چشمان بسته و تعادل پویای سالمندان دلالت داشتند، اما برتری نتایج آزمون تعادل ایستا با چشمان باز در ساعت 16 (8/37 ثانیه) نسبت به ساعت 8 (6/25 ثانیه) معنادار بود (0/018=P). نتیجه‌گیری: براساس یافته‌های پژوهش حاضر، در مورد وقت بهینه انجام تمرینات تعادلی، پیشنهادی وجود ندارد، اما سالمندان بهتر است در حدود ساعت 8 به عوامل خارجی افتادن نظیر ناهمواری سطح زمین توجه بیشتری داشته باشند

Title: بررسی عملکرد سیستم‌های دهلیزی حسی و عمقی و بینایی در کنترل پوسچر مردان سالمند

http://salmandj.uswr.ac.ir/article-1-854-fa.html

اهداف: بررسی عملکرد سیستم‌های حسی به‌عنوان منابع بازخوردی در کنترل تعادل بدن به‌هنگام مواجهه سالمندان با آشفتگی‌های ناگهانی، به دانشمندان در شناخت سازوکار کنترل پوسچر در این گروه سنی و تنظیم برنامه‌های توانبخشی برای آنها کمک می‌کند. هدف این مطالعه بررسی عملکرد سیستم‌های دهلیزی، حسی عمقی و بینایی در کنترل پوسچر مردان سالمند بود. مواد و روش‌ها: نوسانات پوسچر 40 آزمودنی مرد، در دو گروه آ (50تا59سال) و ب (50 تا 69 سال)، در پنج وضعیت طبیعی (I)، آشفتگی دهلیزی (II)، آشفتگی حسی-عمقی (III)، آشفتگی بینایی (IV) و ارائه بازخورد بینایی ویژه از مرکز فشار پا (V) به‌وسیله دستگاه تعادل‌سنج دینامیکی اندازه‌گیری شد و اطلاعات به‌دست‌آمده به روش آماری MANOVA مورد تجزیه و تحلیل قرار گرفت. یافته‌ها: نتایج نشان داد بین عامل سن و نوسانات پوسچر تاثیرمتقابل وجود دارد (0/013=P)، درحالت (I) نوسانات کلی پوسچر گروه ب نسبت به گروه آ بطور معناداری بیشتر بود (0/01=P). ایجاد وضعیت (II) در هر دو گروه، باعث افزایش 1/3 برابر نوسانات کلی پوسچر نسبت به حالت (I) شد. در حالت (III) این افزایش در گروه آ 1/7 برابر و در گروه ب 1/8 برابر شد. در حالت (IV)، نوسانات کلی پوسچر نسبت به حالت (I) در گروه آ، 2/3 برابر و در گروه ب، 2/9 برابر شد. در حالت (V)، نوسانات کلی پوسچر نسبت به حالت (I) در هر دو گروه تغییر معناداری نکرد (0/074=P). همچنین در هر دو گروه، نوسانات پوسچر در جهت قدامی خلفی به‌طور معناداری بیشتر از جهت داخلی جانبی بود (0/031=P). نتیجه‌گیری: با ورود افراد به دهه ششم عمر، به‌علت افول منابع بازخوردی و تضعیف سیستم‌های حسی، اختلالات چشم‌گیری در را‌ه‌اندازی دستورات اصلاحی در سازوکار کنترل پوسچر رخ می‌دهد. در نتیجه نوسانات پوسچر، به‌ویژه در جهت قدامی خلفی، به‌طور قابل توجهی افزایش می‌یابد؛ به‌گونه‌ای که کنترل پوسچر افراد در این سنین تضعیف می‌شود و احتمال زمین‌خوردن آنها قوت می‌گیرد. ضعف مشهودتر سالمندان در برابر اختلالات بصری، نشانگر تکیه ترجیحی آنها بر اطلاعات این سیستم به‌عنوان سیستم غالب درکنترل پوسچر است.

Title: تأثیر تمرینات تعادلی قدرتی و ترکیبی بر تعادل زنان سالمند

http://salmandj.uswr.ac.ir/article-1-726-fa.html

اهداف: سالمندی با کاهش ظرفیت فیزیولوژیکی و عملکردی در ارتباط است که می‌تواند باعث افزایش ناتوانی، کاهش تعادل و افتادن شود؛ بنابراین، هدف این تحقیق بررسی تفاوت تأثیر تمرینات تعادلی، قدرتی و ترکیبی (تعادلی و قدرتی) بر شاخص‌های کنترل قامت (کلی، قدامی-خلفی و میانی-جانبی) زنان سالمند است. مواد و روش‌ها:  24 زن سالمند بدون سابقه آسیب در اندام‌های تحتانی، داوطلبانه در این مطالعه شرکت داشتند که به‌صورت تصادفی در 3 گروه قرار گرفتند: تعادلی (8 نفر)، قدرتی (8 نفر) و ترکیبی (8 نفر). برنامه تمرینی در 18 جلسه در دانشگاه شهیدبهشتی اجرا و نمرات تعادل با استفاده از دستگاه تعادل‌سنج بایودکس اندازه‌گیری شد. تحلیل داده‌ها با آزمون تحلیل واریانس عاملی (3 گروه در 2 زمان)، تحلیل واریانس یک‌طرفه و آزمون تعقیبی بونفرونی (0/05≥P) صورت پذیرفت. یافته‌ها: بعد از تمرینات، گروه تعادلی و ترکیبی نسبت به پیش‌آزمون در شاخص‌های کنترل قامت (کلی، قدامی-خلفی و میانی-جانبی) و گروه تمرین قدرتی در شاخص‌های (قدامی-خلفی و میانی-جانبی) پیشرفت معنی‌داری داشتند. در تعادل کلی بین گروه‌های تعادلی (P=0/0001) و ترکیبی (P=0/001) با گروه قدرتی، تفاوت معنی‌داری وجود داشت. همچنین در تعادل قدامی-خلفی بین گروه‌های تعادلی (P=‌0/0001) و ترکیبی (P=‌0/0001) با گروه قدرتی تفاوت معنی‌داری وجود داشت، اما در تعادل میانی-جانبی بین گروه‌ها تفاوت معنی‌داری وجود نداشت. نتیجه‌گیری: برنامه‌های تمرینی تعادلی باعث اختلال تعادل فرد در حین تمرین و تمرینات ترکیبی باعث کاهش بیشتر نوسان قامتی نسبت به تمرینات قدرتی می‌شود و خطر افتادن را در افراد سالمند کاهش می‌دهد.

Title: اثربخشی تمرین‌ درمانی در آب بر کیفیت زندگی زنان مبتلا به استئوآرتریت زانو

http://salmandj.uswr.ac.ir/article-1-734-fa.html

اهداف: امروزه شرکت در فعالیت بدنی در بهبود عوارض بیماری‌های مزمنی همچون استئوآرتریت پیشنهاد می‌شود. هدف پژوهش حاضر، بررسی تأثیر یک دوره تمرین ‌درمانی در آب بر کیفیت زندگی زنان مبتلا به استئوآرتریت زانو بود. مواد و روش‌ها: این مطالعه از نوع معادل‌سازی متقابل بود و با روش تصادفی ساده، 30 زن مبتلا به بیماری استئوآرتریت زانو (40 تا 74 سال) در دو گروه تجربی قرار گرفتند. از پرسش‌نامه پیامدهای استئوآرتریت و صدمات زانو در سه مرحله پیش، پس و میان آزمون برای اندازه‌گیری تأثیر تمرین ‌درمانی در آب، از نظر بیماران استفاده شد. این پرسش‌نامه 5 عامل درد، عوارض مربوط به بیماری، فعالیت‌های روزمره زندگی، فعالیت‌های ورزشی و عملکردی و کیفیت زندگی را اندازه‌گیری می‌کند. در هشت هفته اول (بین پیش و میان‌آزمون‌)، گروه اول دوره بی‌تمرینی و گروه دوم دوره تمرین خود را داشتند. در هشت هفته دوم (بین میان و پس‌آزمون)، وضعیت تمرینی گروه‌ها معکوس شد. به این ترتیب، برنامه تمرینی هر دو گروه معادل،‌ ولی دوره‌بندی تمرین آنها معکوس بود. تحلیل آماری از طریق تحلیل واریانس یک‌طرفه اندازه‌های مکرر و نسخه 16 نرم‌افزار SPSS انجام شد. یافته‌ها: نتایج دو گروه تجربی مشابه و مؤید یکدیگر بود و نشان داد که بی‌تمرینی با عدم تغییر در تمامی عوامل پرسش‌نامه همراه بود و تمرین موجب پیشرفت عوامل پرسش‌نامه و قطع تمرین باعث پسرفت آن‌ها شد. این یافته با یافته‌های مربوط به اندازه‌گیری‌های عملکردی همسو بود (0/000=P). نتیجه‌گیری: نتایج نشان داد که  برنامه به‌کاررفته در تحقیق حاضر برای مبتلایان به استئوآرتریت زانو مفید بوده و موجب ارتقای کیفیت زندگی آن‌ها شده است.

Title: مقایسه نیم‌رخ عضلانی اسکلتی افراد سالمند با سابقه و بدون سابقه افتادن

http://salmandj.uswr.ac.ir/article-1-690-fa.html

اهداف: در پژوهش حاضر نیم‌رخ عضلانی–اسکلتی افراد سالمند با و بدون سابقه افتادن مقیم آسایشگاه‌های شهرستان رشت مورد مقایسه قرار گرفت. مواد و روش‌ها: آزمودنی‌های این مطالعه شامل 54 نفر از سالمندان زن و مرد 60 سال و بالاتر بود. به‌طور کلی 16 نفر به‌عنوان سالمندان باسابقه افتادن در طول یک سال اخیر با میانگین±انحراف معیار سنی 9/90±79 سال، قد 0/103±1/51 متر و وزن 11/12±60/03 کیلوگرم و 38 نفر به‌عنوان سالمندان بدون سابقه افتادن با میانگین±انحراف معیار سنی 8/16±70/16 سال و قد 0/11±1/56 متر و وزن 14/93±65/6 کیلوگرم در پژوهش شرکت کردند. متغیرهای قوس کف پا، فاصله دو کندیل ران، کایفوز، لوردوز، انعطاف‌پذیری و توانایی راه‌رفتن به‌ترتیب به‌وسیله خط‌کش، کولیس، خط‌کش منعطف، آزمون خمش و رسش و پرسش‌نامه ارزیابی عملکردی راه‌رفتن اندازه‌گیری شد. برای تجزیه و تحلیل داده‌ها از نرم‌افزار SPSS و آزمون تی مستقل استفاده شد و سطح معناداری در این مطالعه 0/05 در نظر گرفته شد. یافته‌ها: در گروه سالمندان باسابقه افتادن میانگین و انحراف معیار متغیرها بدین ترتیب بود: شاخص توده بدنی 3/35±25/95 کیلوگرم بر مترمربع، قوس کف پا 4/34±9/94 میلی‌متر، انعطاف‌پذیری 6/38±8/25 سانتی‌متر، لوردوز 9/48±20/86 درجه، کایفوز 14/98±35/07 درجه. در گروه سالمندان بدون سابقه افتادن نیز میزان این متغیرها به‌شرح ذیل بود: شاخص توده بدنی 5/12±26/87 کیلوگرم بر متر‌مربع، قوس کف پا 5/60±10/95 میلی‌متر، انعطاف‌پذیری 5/24±8/16 سانتی‌متر، لوردوز 14/78±26/66 درجه و کایفوز 9/84±41/11 درجه بود. این دسته از متغیرها در دو گروه تفاوت معناداری نداشتند، ولی در آزمون‌های ارزیابی عملکردی راه‌رفتن و فاصله دو کندیل ران، تفاوت معنادار مشاهده شد. در گروه سالمندان باسابقه افتادن، فاصله دو کندیل ران و آزمون ارزیابی عملکردی راه‌رفتن به‌ترتیب 2/38±2/74 سانتی‌متر و 7/09±12/50 و در گروه سالمندان بدون سابقه افتادن، فاصله دو کندیل ران و آزمون عملکردی راه‌رفتن به‌ترتیب 1/4±1/20 سانتی‌متر و 5/49±23/63 به‌دست آمد. بر این اساس، در گروه سالمندان باسابقه افتادن، فاصله دو کندیل ران بیشتر و سطح عملکرد پایین‌تر بود (05/0≥P). نتیجه‌گیری: یافته‌ها نشان داد که هیچ‌یک از متغیرهای ارتفاع قوس پا، انعطاف‌پذیری، لوردوز و کایفوز در دو گروه مورد مطالعه با هم اختلاف آماری معناداری نداشته‌اند؛ در‌حالی‌که دو گروه در فاصله دو کندیل ران و عملکرد راه‌رفتن با هم اختلاف معناداری داشته‌اند. به‌نظر می‌رسد پرسشنامه عملکردی راه‌رفتن و اندازه‌گیری فاصله دو کندیل ران بهترین عوامل برای تفکیک دو گروه بوده است؛ بنابراین، شاید بتوان از این روش‌ها به‌عنوان روشی مناسب در پیش‌بینی و به‌دنبال آن پیشگیری از افتادن سالمندان استفاده کرد.

Title: مقایسه مثلث ‌شناختی در سالمندان ساکن منزل و آسایشگاه

http://salmandj.uswr.ac.ir/article-1-749-fa.html

اهداف: خطاهای شناختی و نگرش منفی نسبت به خود، دنیا و آینده از مشکلات گسترده سالمندان است که موجب افت کارآیی، کیفیت زندگی و افزایش مرگ‌ومیر می‌شود. این پژوهش، با هدف مقایسه مثلث ‌شناختی (نگرش منفی نسبت به خود، دنیا و آینده) در سالمندان ساکن منزل و آسایشگاه انجام گردید. مواد و روش‌ها: مطالعه حاضر از نوع علّی-مقایسه‌ای بود که تعداد 40 سالمند (20 نفر ساکن منزل و 20 نفر ساکن آسایشگاه) به روش نمونه‌گیری دردسترس انتخاب شدند. پرسش‌نامه مثلث‌ شناختی (CTI) در مورد سالمندان اجرا گردید و داده‌های پژوهش با استفاده از تحلیل واریانس چندمتغیره (MANOVA) مورد تجزیه‌وتحلیل قرار گرفت. یافته‌ها: میانگین سنی افراد مورد مطالعه 16±68 سال بود. یافته‌ها نشان داد بین سالمندان ساکن آسایشگاه و منزل در نگرش نسبت به خود (0/001>P و 229/87=F) نگرش نسبت به دنیا (0/001>P و 138/41=F) و نگرش نسبت به آینده (0/001>P و 151/62=F) تفاوت معنی‌داری وجود دارد. نتیجه‌گیری: اختلال شناختی در سالمندان مقیم سرای سالمندان به‌طور معنی‌داری بیشتر از سالمندان ساکن منزل بود؛ بدین معنا که نگرش سالمندان ساکن منزل در مورد خود، دنیا و آینده در مقایسه با سالمندان ساکن آسایشگاه بهتر بوده است. نتیجه کاربردی که از این مطالعه و با کمک مطالعات آتی مداخله‌ای به‌دست می‌آید این است که توانبخشی شناختی می‌تواند به‌عنوان درمانی برای نگرش منفی سالمندان مطرح گردد.

Title: ارتباط سبک زندگی و سلامت عمومی سالمندان شهر تهران

http://salmandj.uswr.ac.ir/article-1-830-fa.html

اهداف: ارتقای بهداشت و تأمین سلامت سالمندان جامعه -که در سال‌های آتی بخش بزرگی از جمعیت را به خود اختصاص خواهند داد-، از ارکان مهم پیشرفت جوامع است. شواهد موجود نشان می‌دهد بالغ بر 53درصد مرگ‌ومیر افراد، با سبک زندگی آنها ارتباط دارد. اهمیت سبک زندگی و عوامل مرتبط با آن، محققان را برآن داشت که به بررسی سبک زندگی و سلامت بپردازند. هدف کلی این مطالعه تعیین ارتباط سبک زندگی با سلامت عمومی سالمندان شهر تهران بود. مواد و روش‌ها: این مطالعه از نوع توصیفی-تحلیلی بود و جامعه آماری آن را کلیه سالمندان شهر تهران که فاقد هرگونه مشکلات شناختی بودند، تشکیل می‌دادند. تعداد نمونه 400 نفر سالمند بالای 60 سال بودند که از 5 محدوده تهران (شمال، جنوب، مرکز، شرق و غرب) به‌صورت تصادفی-خوشه‌ای چندمرحله‌ای انتخاب و وارد مطالعه شدند. ابزار جمع‌آوری اطلاعات پرسش‌نامه مشتمل بر سه بخش دموگرافیک، سبک زندگی سالمندان در ابعاد (ورزش، تغذیه، روابط بین فردی و اجتماعی، پیشگیری و مدیریت استرس) و سلامت عمومی 28-GHQ موردی بود که از طریق مصاحبه تکمیل گردید. یافته‌ها: یافته‌های پژوهش نشان داد که سبک زندگی 95درصد سالمندان تهران در حد متوسط قرار دارد و نتایج تحلیل با رگرسیون چندگانه نشان داد که سبک زندگی و حیطه‌های آن با سلامت عمومی و زیرمقیاس‌های آن رابطه‌ای معنادار (0/01>P) و نمره سبک زندگی نیز با نمره سلامت عمومی، رابطه معنادار معکوس در حد متوسط داشت (0/4-=r). نتیجه‌گیری: با توجه به یافته های پژوهش مبنی بر رابطه بین سلامت عمومی و سبک زندگی، توصیه می‌شود سیستم‌های بهداشتی و مسئولین امر، برنامه‌ریزی‌های دقیق‌تری برای ارتقای سبک زندگی و سلامت روانی سالمندان انجام دهند. اگرچه نتایج مطالعه نشان داد که  در سالمندان فقط 40درصد از تغییرات سلامت عمومی تحت‌تأثیر سبک زندگی قراردارد که  این بدان معنی است که سلامت عمومی با متغیرهای دیگری غیر از سبک زندگی نیز در ارتباط است.

Title: وضعیت نشانگان بازنشستگی در بازنشستگان

http://salmandj.uswr.ac.ir/article-1-745-fa.html

اهداف: بازنشستگی بر ابعاد مختلف جسمی، روانی، اجتماعی، اقتصادی و معنوی فرد تأثیرگذار است. ازآنجاکه لازم است عواملی که موجب ارتقا یا تضعیف نشانه‌های سلامت عمومی در بازنشستگان می‌شود، مورد توجه جدی قرار گیرد، شناسایی این عوامل بسیار مهم است؛ بنابراین، هدف این مطالعه بررسی وضعیت نشانگان بازنشستگی در بازنشستگان شهر بابل بوده است. مواد و روش‌ها: این مطالعه مقطعی روی 205 نفر از بازنشستگان بالای 50 سال انجام شد. داده‌های پژوهش توسط پرسش‌نامه‌‌ای مشتمل بر دو بخش اطلاعات دموگرافیک و نشانگان بازنشستگی جمع‌آوری شد. پرسش‌نامه نشانگان بازنشستگی شامل 40 سؤال به‌صورت پنج گزینه (هرگز تا همیشه) روی چهار احساس درماندگی و شکست، احساس پیری و بطالت، احساس تلاش و جهت‌گیری جدید و احساس سردرگمی و تعارض بوده است. سپس داده‌ها وارد نرم‌افزار آماری SPSS شد و مورد تجزیه‌وتحلیل آماری قرار گرفت. برای تعیین همبستگی بین متغیرها از آزمون پیرسون و اسپیرمن و برای تجزیه تحلیل داده‌ها از آزمایه‌های آماری ضریب همبستگی، آزمون تی و تحلیل واریانس یک‌طرفه (آنوا) استفاده گردید. همچنین سطح معنی‌داری آماری در تمام آزمون‌ها 0/05 در نظر گرفته شد. یافته‌ها: افراد شرکت‌کننده در این مطالعه، 53/2درصد مرد و 46/8درصد زن با میانگین سنی 5/87±61/13 سال بودند. میانگین امتیاز احساس درماندگی و شکست 0/56±0/08، احساس پیری و بطالت 0/51±3/73، احساس تلاش و جهت‌گیری جدید 0/65±3/47 و احساس سردرگمی و تعارض 49/56±2/0 به‌دست آمد. احساس درماندگی و شکست با احساس سردرگمی و تعارض همبستگی مستقیم داشت (0/0001>P). همچنین احساس تلاش و جهت‌گیری جدید با احساس پیری و بطالت، همبستگی مستقیم و با احساس درماندگی و شکست همبستگی معکوس داشت (هر دو 0/0001>P). نتیجه‌گیری: نتایج طرح نشان داد که کمابیش بازنشستگان شهرستان بابل از نشانگان بازنشستگی رنج می‌رند؛ بنابراین، توجه به این قشر و بهبود شرایط افراد بازنشسته ضروری به‌نظر می‌رسد.

Title: رابطه حمایت اجتماعی و بهزیستی روانی در سالمندان مراجعه‌کننده به مراکز توانبخشی روزانه

http://salmandj.uswr.ac.ir/article-1-953-fa.html

اهداف: سالمندان یکی از گروه‌های آسیب‌پذیر جامعه هستند که از نظر جسمی، روانی، اجتماعی و بهداشتی نیازهای ویژه‌ای دارند. سالمندشدن و به‌دنبال آن افزایش بیماری‌های مزمن، بیشتر باعث افزایش معلولیت، وابستگی و ناتوانی سالمندان جامعه می‌شود. با توجه به اینکه انواع حمایت اجتماعی در افزایش سلامت روانی سالمندان از اهمیت خاصی برخوردار است، پژوهش حاضر به بررسی رابطه بین حمایت اجتماعی با بهزیستی روانی در سالمندان مراکز جامع توانبخشی روزانه سالمندان استان تهران، می‌پردازد. مواد و روش‌ها: این مطالعه از نوع همبستگی و جامعه پژوهش آن را کلیه سالمندان مراجعه‌کننده به مراکز جامع توانبخشی روزانه استان تهران در سال 1393 تشکیل می‌دادند که 147 نفر از آنان به روش نمونه‌گیری خوشه‌ای-تصادفی انتخاب شدند. در این مطالعه، برای جمع‌آوری داده‌ها از پرسش‌نامه حمایت اجتماعی نوربک و برای ارزیابی بهزیستی روانی از پرسش‌نامه و مقیاس سنجش روحیه فیلادلفیا استفاده گردید و داده ها به‌وسیله نسخه 20 نرم‌افزار SPSS تجزیه‌وتحلیل شدند. یافته‌ها: در این مطالعه 147 سالمند شرکت کردند که80 نفر (54/4درصد) از آنان مرد بودند. میانگین سنی پاسخ‌گویان در این پژوهش 71/9سال (انحراف معیار 5/09) بود. براساس یافته‌ها، میانگین بهزیستی روانی (میانگین=8/65) (انحراف معیار 2/28) بود که نشان‌دهنده وضعیت متوسط بهزیستی روانی است. علاوه‌براین نتایج نشان داد که بین حمایت اجتماعی ادراک‌شده (P=0/002) با بهزیستی روانی، رابطه معنادار و مثبت وجود دارد. نتیجه‌گیری: براساس یافته‌های این مطالعه، حمایت اجتماعی با بهزیستی روانی رابطه مثبت دارد. بنابراین، افزایش حمایت اجتماعی سالمندان می‌تواند تأثیر مهمی بر بهزیستی روانی و عملکرد اجتماعی سالمندان داشته باشد. از سوی دیگر، افزایش حمایت‌های اجتماعی نیز می‌تواند باعث تقویت بهزیستی روانی سالمندان شود.

Title: طراحی و روان‌سنجی پرسش‌نامه خودمراقبتی سالمندان

http://salmandj.uswr.ac.ir/article-1-693-fa.html

اهداف: هدف مطالعه حاضر، طراحی ابزار اختصاصی متناسب با جامعه ایرانی برای بررسی خودمراقبتی برای سالمندان بود. مواد و روش‌ها: مطالعه حاضر، پژوهشی روش‌شناختی است که در 2 مرحله انجام گرفت. در مرحله اول گویه‌ها و طبقات اولیه ابزار جدید براساس مروری بر مطالعات منطبق بر «نظریه اورم» استخراج شد. مرحله دوم ویژگی‌های روان‌سنجی پرسش‌نامه با بهره‌گیری از روایی صوری، روایی محتوا، روایی سازه، همسانی درونی مورد بررسی قرارگرفت. در مرحله اخیر، 225 سالمند ارومیه‌ای به‌صورت تصادفی از خانه‌های سالمندان و کانون‌های جهان‌دیدگان منتخب شهر ارومیه، انتخاب شدند و در مطالعه شرکت کردند. یافته‌ها: 45 گویه پرسش‌نامه، براساس مروری بر مطالعات منطبق بر نظریه اورم استخراج شد. پس از تعیین روایی صوری و محتوا به‌صورت کمّی و کیفی، عبارات پرسش‌نامه به 40 عبارت کاهش یافت. نتیجه تحلیل عاملی اکتشافی در این پرسش‌نامه، بیانگر وجود 5 عامل بود. این 5 عامل درمجموع 79/93درصد از واریانس کل آزمون را تبیین می‌نمود. محاسبه ضریب آلفای کرونباخ مؤید همسانی درونی بالای پرسش‌نامه (0/864) بود. نتیجه‌گیری: یافته‌های نهایی این پژوهش حاکی از آن است که پرسش‌نامه حاضر با عنوان «پرسش‌نامه بررسی توانایی خودمراقبتی سالمندان» با 40 عبارت، با وجود ویژگی‌هایی نظیر طراحی ابزار براساس مروری بر مطالعات منطبق بر نظریه اورم، نمره‌گذاری ساده، پایایی و روایی مناسب و قابلیت به کارگیری در موقعیت‌های مختلف توسط ارائه‌دهندگان خدمات بهداشتی، ازجمله پرستاران، ابزاری مناسب برای بررسی توانایی خودمراقبتی سالمندان است.

Title: تأثیر درمان نامیدن بدون خطا بر اختلال نامیدن یک سالمند زبان‌پریش

http://salmandj.uswr.ac.ir/article-1-818-fa.html

اهداف: آسیب نامیدن یکی از رایج‌ترین مشکلاتی است که افراد زبان‌پریش و درمانگران با آن درگیرند. با توجه به تأثیر مخرب آنومی بر کارآیی ارتباطی بیماران، پژوهش حاضر با هدف بررسی اثربخشی درمان نامیدن بدون خطا بر توانایی نامیدن در یک بیمار زبان‌پریش صورت پذیرفت. مواد و روش‌ها: آزمودنی به روش نمونه‌گیری هدفمند انتخاب شد. این پژوهش مداخله‌ای تک‌آزمودنی با طرح خط پایه-درمان-خط پایه، روی خانمی 69 ساله که به زبان‌پریشی بروکا با شدت متوسط و خطاهای واجی مبتلا بود، انجام شد. شرکت‌کننده باسواد، راست دست و فارسی‌زبان بود و مشکلات حرکتی شدید و نقایص درک شنیداری قابل توجهی نداشت. بیمار به‌مدت 10 جلسه با روش نامیدن بدون خطا از طریق قرارداد درمانی رایمر و همکاران (2012) تحت درمان قرار گرفت. در این روش به بیمار فرصت‌های متعددی برای تمرین نام صحیح تصویر از طریق تکرار و خواندن شفاهی داده شد، در‌حالی‌که از بروز خطا حین آموزش اجتناب می‌شد. یافته‌ها: در این بیمار اندازه اثر در تکلیف نامیدن در مواجهه پس از ارائه مداخله به‌ترتیب برای کلمات آموزشی و کنترل 5/42 و 2/38 به‌دست آمد. شیب تغییرات درصد پاسخ‌های صحیح به‌ترتیب برای کلمات آموزشی و کنترل 7/6 و 4/15 بود. پیشرفت در نامیدن کلمات آموزشی و کنترل که یک هفته بعد بررسی گردید، همچنان در سطح بالاتر از خط پایه حفظ شد. همچنین در نمرات آزمون‌های زبان‌پریشی و نامیدن پس از درمان پیشرفت دیده شد. نتیجه‌گیری: بر اثر درمان نامیدن بدون خطا در این پژوهش،  بیمار در  نامیدن اسامی مجموعه آموزشی پیشرفت معناداری نشان داد، اما تعمیم بهبودی به کلمات کنترل چشم‌گیر نبود. می‌توان این یافته‌ها را این‌گونه تعبیر کرد که ممکن است این روش به‌عنوان یک قرارداد درمانی در دیگر بیماران زبان‌پریش فارسی‌زبان دارای آنومی نیز مفید باشد

Title: بررسی کیفیت زندگی، افسردگی و فعالیت‌های روزمره زندگی در سالمندان روستایی شهرستان بیرجند سال 1391

http://salmandj.uswr.ac.ir/article-1-753-fa.html

اهداف: با وجود پیشرفت‌های علمی انجام‌شده در حوزه پزشکی و افزایش امید به زندگی در جوامع امروزی، شاهد رشد قابل توجه جمعیت سالمند در ده‌های اخیر در کشور بوده‌ایم. سالمندان با مخاطرات مختلف و متفاوت از دیگر گروه‌های سنی مواجه هستند. این مطالعه به‌منظور بررسی کیفیت زندگی، افسردگی و فعالیت‌های روزمره زندگی در سالمندان روستایی شهرستان بیرجند در سال 1391 انجام شد. مواد و روش‌ها: این مطالعه توصیفی-تحلیلی از نوع مقطعی روی 360 سالمندان روستایی شهرستان بیرجند صورت پذیرفت. ابزار جمع‌آوری اطلاعات پرسش‌نامه 26 سؤالی استاندارد کیفیت زندگی سازمان جهانی بهداشت، پرسش‌نامه 15 سؤالی افسردگی سالمندان (GDS)، پرسش‌نامه 6 سؤالی انجام فعالیت‌های روزمره زندگی (ADL) و پرسش‌نامه 7 سؤالی انجام فعالیت‌های روزمره زندگی با وسیله (IADL) بوده است. داده‌های پژوهش با استفاده از نسخه 19 نرم‌افزار SPSS مورد تجزیه و تحلیل قرار گرفت و مقادیر 0/05≥P معنادار در نظر گرفته شد. یافته‌ها: در این پژوهش، میانگین و انحراف معیار کیفیت زندگی برابر با 10/4±77/9 و میانگین و انحراف معیار افسردگی 2/9±5/5، میانگین و انحراف معیار فعالیت‌های روزمره زندگی بدون وسیله 1/5±6/5 و میانگین و انحراف معیار فعالیت‌های روزمره زندگی با وسیله (3±12) محاسبه گردید. از میان متغیرهای دموگرافیک تحرک بدنی و ورزش، تحصیلات، وضع تأهل، درجات افسردگی و فعالیت‌های روزمره با و بدون وسیله با کیفیت زندگی اختلاف معنی‌دار داشت (0/05≥P). همچنین، بین کیفیت زندگی و افسردگی رابطه معنی‌دار و معکوس قوی (0/56-=R)، بین فعالیت‌های روزمره زندگی بدون وسیله (0/32-=R) و با وسیله (0/39-=R) کیفیت زندگی رابطه معنی‌دار و معکوس مشاهده شد. نتیجه‌گیری: نتایج این مطالعه نشان داد فعالیت‌های روزمره زندگی، باعث افزایش کیفیت زندگی و کاهش افسردگی می‌شود. براساس این نتایج، مسئولان سیستم خدمات بهداشتی و درمانی ضمن برنامه‌ریزی مناسب برای اطلاع‌رسانی فواید فعالیت در دوره سالمندی، باید برای ایجاد محیط‌ها و برنامه‌های متناسب با فعالیت این قشر در شهر و کشور اقدامات لازم را انجام دهند.

Title: مقایسه تأثیر 8 هفته تمرین هوازی و تمرین مقاومتی بر سطح عامل نوروتروفیک مشتق از مغز سرم مردان سالمند

http://salmandj.uswr.ac.ir/article-1-814-fa.html

اهداف: عامل نوروتروفیک مشتق از مغز سرم (BDNF) یکی از عواملی است که در دوران سالمندی در اثر کمبود فعالیت کاهش می‌یابد؛ از‌این‌رو، هدف از اجرای این پژوهش مقایسه اثر 8 هفته تمرین هوازی و تمرین مقاومتی روی سطوح عامل نوروتروفیک مشتق از مغز سرم در مردان سالمند بود. مواد و روش‌ها: در این تحقیق نیمه‌تجربی، 30 مرد سالمند با میانگین سن 5±70 از کانون جهان‌دیدگان انتخاب و به‌طور تصادفی در دو گروه تجربی و یک گروه کنترل تقسیم شدند. از کلیه آزمودنی‌ها 24 ساعت قبل از شروع تمرین و 24 ساعت پس از پایان دوره آزمون، به‌صورت ناشتا خون گرفته شد. گروه‌های تجربی به‌مدت 8 هفته، هر هفته 3 جلسه تمرین هوازی به‌مدت 45-30 دقیقه با شدت 75-60 درصد ضربان قلب بیشینه و تمرین مقاومتی به‌مدت 40-30 دقیقه و با 60-40 درصد یک تکرار بیشینه به تمرین پرداختند و گروه کنترل در این مدت در هیچ برنامه تمرینی شرکت نکردند. نتایج به‌دست‌آمده با استفاده از نسخه 19 نرم‌افزار SPSS تجزیه و تحلیل شده است. یافته‌ها: میانگین و انحراف معیار قبل و بعد از 8 هفته تمرین در گروه تمرین هوازی به‌ترتیب 66/95±174/3، 116/7±286/7 و در گروه تمرین مقاومتی 7/03±217/7 و 143/1±313/2 و در گروه کنترل 49/04±112/6 و 47/8±118/3 بود. بنابراین، سطوح عامل نوروتروفیک مشتق از مغز سرم در هر دو گروه‌های تجربی در مقایسه با گروه کنترل به‌طور معنی‌داری افزایش یافت (P≤0/05). نتیجه‌گیری: نتایج این تحقیق نشان می دهد که تمرینات هوازی و مقامتی هر دو سبب افزایش سطوح عامل نوروتروفیک مشتق از مغز سرم می‌شوند.

Title: تأثیر یک دوره تمرین مقاومتی بر غلظت فاکتور رشد اندوتلیال عروقی در زنان سالمند

http://salmandj.uswr.ac.ir/article-1-853-fa.html

اهداف: سلول های اندوتلیال عروقی نقش مهمی در تنظیم فعالیت‌های عروقی با تولید مواد فعال‌ننده عروق، مانند فاکتور رشد اندوتلیال عروقی برعهده دارد؛ ازاین‌رو، هدف از تحقیق حاضر تأثیر یک دوره تمرینات مقاومتی بر غلظت فاکتور رشد اندوتلیال زنان سالمند بود. مواد و روش‌ها: در این تحقیق نیمه‌تجربی، 24 زن یائسه با میانگین و انحراف معیار سن 6/02±67/37 سال، قد 8/12±153/02 سانتی‌متر، وزن 12/03±65/78 کیلوگرم، شاخص توده بدنی 87/16±26/4 (kg/m2)، درصد چربی 3/65±18/61 و نسبت دور کمر به شکم 0/4±0/92 سانتی‌متر به‌صورت هدفمند انتخاب و به‌طور تصادفی به دو گروه 12 نفره کنترل و تجربی تقسیم شدند. گروه تجربی هشت هفته تمرین مقاومتی، سه جلسه در هفته با شدت 40 تا 65درصد یک تکرار بیشینه و با اضافه بار 5درصد بعد از هر 6 جلسه را انجام داد. قبل و بعد از 8 هفته تمرین، سطح استراحتی غلظت فاکتور رشد اندوتلیال عروقی اندازه‌گیری و ثبت شد. برای تجزیه و تحلیل داده‌ها با استفاده از آمار توصیفی و استنباطی نظیر تی همبسته و تی مستقل استفاده شد. سطح معنی‌داری کمتر از 0/05 در نظر گرفته شد. یافته‌ها: میانگین و انحراف معیار قبل و پس از هشت هفته غلظت فاکتور رشد اندوتلیال عروقی در گروه کنترل به‌ترتیب 3/62±25/61، 2/88±25/72 و در گروه تجربی به‌ترتیب 4/48±26/48، 2/81±23/81 بود؛ بنابراین، اختلاف میانگین فاکتور رشد اندوتلیال عروقی در پیش‌آزمون و پس‌آزمون گروه تجربی معنی‌دار بوده است (0/015=P) . در‌حالی‌که در گروه کنترل 0/862=P معنادار نبوده است. نتیجه‌گیری: نتایج این تحقیق نشان داد یک دوره تمرین مقاومتی بر کاهش غلظت فاکتور رشد اندوتلیال عروقی پلاسمای زنان سالمند تأثیر معنی‌داری دارد.

Title: مقایسه سطح مشارکت اجتماعی سالمندان استفاده‌کننده از وسایل کمک‌تحرکی با سالمندان بدون وسیله کمک‌تحرکی

http://salmandj.uswr.ac.ir/article-1-839-fa.html

اهداف: مشارکت اجتماعی از اجزای مهم سلامت در فرایند سالمندی و به‌عنوان بخشی از زندگی و یکی از اجزای کلیدی عملکرد به‌شمار می‌رود که باعث بهبود سلامت فیزیکی و روانی سالمندان می‌شود. این احتمال وجود دارد که استفاده از وسیله کمک تحرکی روی مشارکت اجتماعی سالمندان تأثیر گذارد. هدف از مطالعه حاضر مقایسه وضعیت مشارکت اجتماعی سالمندان دارای وسیله کمک تحرکی با سالمندان بدون وسیله کمک تحرکی در شهر تهران است. مواد و روش‌ها: در این پژوهش مقطعی، سالمندان بدون وسیله کمک‌تحرکی با سالمندانی که از وسیله کمک‌تحرکی استفاده می‌کنند، از نظر سطح مشارکت اجتماعی مقایسه شدند. 79 سالمند (39 زن و 40 مرد) 60 سال و بالاتر به روش نمونه‌گیری دردسترس انتخاب و پرسشنامه‌های عادات زندگی، ارزیابی مختصر شناختی و پرسش‌نامه اطلاعات فردی را تکمیل شدند. به‌منظور تجزیه و تحلیل اطلاعات برای داده‌های پیوسته دارای توزیع طبیعی از آزمون‌های تی و آنوا و برای داده‌های گسسته یا با توزیع غیرطبیعی از آزمون‌های کروسکال‌والیس و من-ویتنی استفاده گردید. یافته‌ها: براساس نتایج تحقیق، سالمندانی که از وسیله کمک‌تحرکی استفاده می‌کردند، مشارکت اجتماعی کمتری در مقایسه با سالمندان بدون وسیله کمک‌تحرکی داشتند (P<0/05). سالمندان گروه سنی 74-60 سال سطح مشارکت اجتماعی بالاتری نسبت به گروه سالمندان 89-75 سال داشتند (P<0/05). از نظر جنسیت و وضعیت تأهل بین سالمندان استفاده‌کننده از وسیله کمک‌تحرکی و بدون وسیله کمک‌تحرکی تفاوتی از نظر سطح مشارکت اجتماعی دیده نشد (P<0/05). نتیجه‌گیری: نوع وسیله کمک‌تحرکی روی مشارکت اجتماعی سالمندان تأثیر می‌گذارد.  نتایج نشان می‌دهد  سالمندان با توانایی فیزیکی بالاتر، مشارکت بهتری دارند. سالمندان دارای وسیله کمک‌تحرکی برای مشارکت با محدودیت‌هایی در جامعه روبه‌رو هستند که لازم است گروه توانبخشی به بهبود مشارکت فرد در جامعه توجه بیشتری نمایند.

Title: اثربخشی آموزش مفاهیم واقعیت‌درمانی بر شادکامی مردان سالمند شهر اردبیل

http://salmandj.uswr.ac.ir/article-1-1025-fa.html

اهداف: پژوهش حاضر با هدف بررسی اثربخشی واقعیت‌درمانی بر شادکامی سالمندان صورت گرفت.   مواد و روش‌ها: مطالعه حاضر از نوع آزمایشی پیش‌آزمون-پس آزمون با گروه کنترل بود. جامعه آماری شامل سالمندان مرد «مرکز ماندگاران شهرستان اردبیل» بود. با استفاده از ملاک‌های ورود و خروج، 50 نفر از سالمندان ارزیابی و از میان آنها،30 نفر واجد شرایط ورود به مطالعه شناخته شدند و به روش تصادفی در دو گروه آزمایش و کنترل قرار گرفتند. مقیاس شادکامی آکسفورد برای دو گروه آزمایش و کنترل اجرا گردید. به‌منظور گردآوری داده­‌ها، از پرسش‌نامه شادکامی آکسفورد (آرگیل، مارتین و گرسلند، 1989) استفاده شد. جلسات آموزش واقعیت‌درمانی برای گروه مداخله، به‌صورت هفته‌­ای دو جلسه و به‌مدت هشت جلسه برگزار و پیگیری یک‌­ماهه نیز انجام شد. تجزیه‌وتحلیل داده­‌ها با استفاده از آزمون تحلیل واریانس با اندازه‌­گیری­‌های مکرر انجام شد. یافته‌ها: نتایج نشان داد تفاوت میانگین نمرات شادکامی دو گروه معنی‌­دار است و واقعیت‌درمانی به‌صورت معنی­‌داری موجب افزایش شادکامی سالمندان می‌شد (0/05>P). همچنین، مقایسه نمرات میانگین پیش‌آزمون و پس‌آزمون در دو گروه، حاکی از افزایش نمرات شادکامی در تمامی خرده‌مقیاس‌­ها در مقطع پس‌آزمون، با اختلاف معنی‌­دار بود (0/001>P). نتیجه‌گیری: نتایج این پژوهش نشان داد واقعیت‌درمانی در افزایش مسئولیت­‌پذیری و افزایش شادکامی سالمندان مفید است. اجرای چنین جلسات گروهی در اقامتگاه‌­ها و مراکز تجمع سالمندان توصیه می‌­شود.

Title: بررسی تأثیر مداخله آموزشی بر ارتقای فعالیت فیزیکی مردان سالمند شهر قم: کاربرد مدل فرانظری

http://salmandj.uswr.ac.ir/article-1-940-fa.html

اهداف: هدف از این مطالعه تعیین تأثیر مداخله آموزشی بر مبنای الگوی فرانظری در ارتقای فعالیت فیزیکی مردان سالمند بود. مواد و روش‌ها: این مطالعه از نوع کارآزمایی بالینی روی 140 نفر از سالمندان عضو کانون‌های بازنشستگان شهر قم با ویژگی‌های انتخاب تصادفی در دو گروه مداخله و کنترل انجام شد. ابزار استفاده‌شده این پژوهش پرسش‌نامه محقق‌ساخته مبتنی‌بر الگوی فرانظری و پرسش‌نامه فعالیت فیزیکی سالمندان «چامپس» بود. برای تحلیل نتایج از آزمون کای-دو و تحلیل واریانس با داده‌های تکراری در نسخه 18  نرم‌افزار SPSS استفاده شد. این مطالعه در پایگاه ثبت کارآزمایی‌های بالینی (IRCT) به شماره IRCT201407208742N5 تأیید شد. یافته‌ها: قبل از آموزش دو گروه مداخله و کنترل، از نظر متغیرهای فردی و وضعیت فعالیت فیزیکی تفاوت معنی‌داری نداشتند (P<0/05). علاوه‌براین، میانگین نمره ساختارهای مربوط به پرسش‌نامه مبتنی‌بر الگو نیز تفاوت معنی‌‌داری پس از آموزش در این گروه داشتند (0/05>P). در گروه کنترل نیز تفاوت معنی‌داری در فعالیت فیزیکی و میانگین نمره ساختارهای پرسش‌نامه وجود نداشت. نتیجه‌گیری: براساس یافته‌های پژوهش، انجام مداخله آموزشی برمبنای الگوی فرانظری در ارتقای فعالیت فیزیکی مؤثر و دارای اعتبار است و الگوی فرانظری می‌تواند در این زمینه مفید باشد. با کاربرد این الگو و شناسایی عوامل تسهیل‌گر و موانع رفتار فعالیت فیزیکی در سالمندان، می‌توان برای ارتقای فعالیت جسمانی منظم گام‌های مؤثری برداشت.

Title: مطالعه ساختاریافته کیفیت زندگی سالمندان در ایران (92-1383)

http://salmandj.uswr.ac.ir/article-1-764-fa.html

اهداف: با توجه به افزایش روزافزون سالمندان، توجه به مسائل آنان از جمله امور مهم در سیاست­‌گذاری اجتماعی کشورها محسوب می­‌شود. بدون آگاهی از نیازهای اساسی و ابعاد سالمندی نمی‌­توان به برنامه­‌ریزی در راستای رفاه روانی و اجتماعی این قشر پرداخت. هدف این پژوهش، بررسی این نیازها و ابعاد با توجه به مقالات علمی منتشرشده در نشریات بین سال‌­های 1383 تا 1392 است. مواد و روش‌ها: برمبنای الگوی مفهومی کیفیت زندگی «هیوجز» (1990)، مطالعه ساختاریافته کیفیت زندگی سالمندان برای بررسی ابعاد و شاخص­‌های کیفیت زندگی در بین 62 مقاله مجلات علمی-پژوهشی منتشرشده و دردسترس در یک بازه 9 ساله انجام گرفت. یافته‌ها: نتایج این پژوهش از برجسته‌بودن بُعد وضعیت سلامتی سالمند در مقالات حکایت دارد (100درصد). مفهوم کیفیت زندگی، مفهومی چندجانبه و چند­بُعدی است که تنها جنبه­‌های فیزیکی و جسمی را دربرنمی‎گیرد و اندازه‎گیری و پی‌بردن به آن، خود در گرو عوامل مختلف فرهنگی، اجتماعی، اقتصادی و حتی محیطی است که کمترین عوامل مورد بررسی، رضایت فردی و عوامل محیط فیزیکی هریک با 11/3درصد است. بیشتر راهکارهای ارائه‌شده برای ارتقا و بهبود کیفیت زندگی سالمندان در ایران در سطح کارکردهای کلان و ساختاری 69/35درصد است که می‌­تواند در سیاست­‌گذاری­‌ها و برنامه‌­ریزی­‌ها مورد استفاده قرار گیرد و راهی در جهت بهبود و ارتقای کیفیت زندگی سالمندان پیشِ‌روی برنامه‌ریزان و سیاست‌گذاران قرار دهد. نتیجه‌گیری: کیفیت زندگی سالمندان در ایران از جمله موارد ضروری است که در صورت توجه به همه ابعاد آن و استفاده از برنامه‌­های مداخله‌ای، آموزشی و حمایتی می­‌تواند بهبود و ارتقا یابد.
